# Robust identification of interactions between heat-stress responsive genes in the chicken brain using Bayesian networks and augmented expression data

**DOI:** 10.1038/s41598-024-58679-3

**Published:** 2024-04-19

**Authors:** E. A. Videla Rodriguez, John B. O. Mitchell, V. Anne Smith

**Affiliations:** 1 School of Biology, University of St Andrews, St Andrews, Fife, KY16 9TH UK; 2 EaStCHEM School of Chemistry and BSRC, University of St Andrews, St Andrews, Fife, KY16 9ST UK

**Keywords:** Bayesian network, Stress, Gene, Chicken, Animal physiology, Computational biology and bioinformatics, Computational models, Data mining, Gene regulatory networks, Machine learning

## Abstract

Bayesian networks represent a useful tool to explore interactions within biological systems. The aims of this study were to identify a reduced number of genes associated with a stress condition in chickens (*Gallus gallus*) and to unravel their interactions by implementing a Bayesian network approach. Initially, one publicly available dataset (3 control vs. 3 heat-stressed chickens) was used to identify the stress signal, represented by 25 differentially expressed genes (DEGs). The dataset was augmented by looking for the 25 DEGs in other four publicly available databases. Bayesian network algorithms were used to discover the informative relationships between the DEGs. Only ten out of the 25 DEGs displayed interactions. Four of them were Heat Shock Proteins that could be playing a key role, especially under stress conditions, where maintaining the correct functioning of the cell machinery might be crucial. One of the DEGs is an open reading frame whose function is yet unknown, highlighting the power of Bayesian networks in knowledge discovery. Identifying an initial stress signal, augmenting it by combining other databases, and finally learning the structure of Bayesian networks allowed us to find genes closely related to stress, with the possibility of further exploring the system in future studies.

## Introduction

The effects of stress on the physiology of poultry species have been widely explored, evaluating the alteration of different indicators^1–5^. Among the consequences of the activation of the stress response, differences in the expression patterns of genes have been identified, driven by the exposure to a stress condition, by using microarray technologies and bioinformatic tools^6–8^. Even though this approach allows the possibility of identifying differentially expressed genes between two possible conditions, the final number of genes might be quite extensive and they can be associated with several aspects of the physiology, such as immunity, neurogenesis, cell signalling, among others^6,9^. Narrowing down sets of differentially expressed genes to ones that interact with each other could provide pointers to key pathways or biological processes that are involved in the stress condition. Consequently, exploring the genetics of the brain under stress conditions might bring new knowledge, with future implications on the health and welfare of poultry species.

Interactions within complex biological systems, such as amongst genes in a stress response, could be further explored by Bayesian networks (BN). BNs can be used for several purposes, including the identification of highly important variables together with their informative relationships and interactions^10,11^. BNs are represented by a directed acyclic graph (DAG), displaying the joint probability distribution of a given set of variables^10,11^. The DAG consists of nodes, representing each one of the given variables, and arcs, representing the statistical relationships between variables^10^. Considering the complexity of genetic systems, BNs allow the possibility of identifying relationships between variables that are not necessarily linear or additive in nature, this being one of the main advantages of using BNs to get further insights into complex systems^10^.

Genetic studies in poultry species involve a relatively small number of individuals, considering that bioinformatic analysis can be used in a small set of samples with the aim of identifying differentially expressed genes between non-stressed and stressed birds. While analysing gene expression one experiment at a time provides the above-mentioned sets of differentially expressed genes, there exists a wealth of information from other experiments that have also collected gene expression data from the same organism and tissue. This data can augment that collected in a particular designed experiment, providing further information on how genes interact. In this context, the aims of this study were to use such an augmented dataset to identify a reduced number of genes associated with a stress condition in chickens (*Gallus gallus*) that interact with each other, unravelling their relationships and interactions by the implementation of a BN approach. Additionally, we present and describe a series of steps to be taken and decisions to be made, that will allow other researchers to analyse their own datasets, especially if they need to collect and augment databases, discretize the data, learn the structure of BNs, and combine multiple searches into one weighted network.

## Results

In order to learn the structure of the BN, we had to take multiple steps and make a series of decisions. Our starting point was to identify a stress signal from a dataset consisting of three chickens raised under control conditions and three chickens raised under heat stress conditions (acute heat stress, temperature 40 ± 1 °C, for 3 h). This stress signal initially consisted of 25 genes that showed differential expression patterns (differentially expressed genes (DEG), see Supplementary Table S1 online), identified by bioinformatic tools (see Methods). Considering that the number of observations was borderline sufficient to learn useful structure of BNs, the number of observations was augmented by searching for the stress signal (represented by the 25 DEGs) in four other datasets sharing the same animal model (the chicken), the same tissue (the brain), and the same high-throughput technology (microarray). Our decision was made by following one of the premises of machine learning and statistical modelling, preferring the simplest model than can explain the data, and hence we select the least complex amongst the experimental designs as our starting point. While here we work with entirely publicly available data, we envision a situation parallel to researchers having a small set of their own pilot data they wish to augment with public databases, thus further supporting choice of the simplest experimental design. The rest of the studies that shared the same three characteristics had extra experimental factors, such as age, sex, and/or breed. Although a small number of observations will not prevent us from learning the structure of a BN, augmenting the data will increase the robustness of the model, in addition to complementing the findings of previous studies instead of focusing on them independently. As a way to deal with the noise associated with the different experimental designs, before combining the datasets into one large dataset, each dataset was individually discretized into three-state discrete variables (see “Methods”). The new dataset consisted of 46 observations and 25 DEGs.

Once the dataset was in place, a BN was learned following the roadmap we have laid out in previous work^21^ to ensure reproducible results. This included use of a Simulated Annealing algorithm, building consensus networks from 100 top-scoring networks, and ultimately identifying those arcs with the most support (present in ≥ 50% of 50 repeated consensus networks) as our resulting BN (see “Methods” for details).

**Figure 1:**
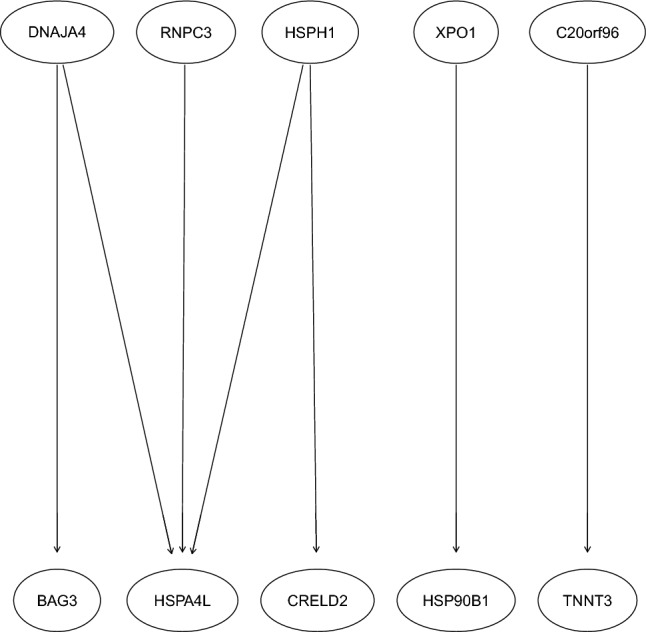
Bayesian network corresponding to highly significant genes related to stress. Nodes correspond to genes, while arcs represent the relationship between genes. The network was built considering the arcs present in at least 25 out of 50 consensus networks. Note that the direction of the arrows does not represent causation, but instead a statistical relationship.

The overall structure of the resulting network is shown in Fig. 1. The initial number of DEGs showing differential expression patterns between the control condition and the heat stress condition was 25, however, the network consisted of only 10 genes out of these initial set of 25 DEGs. Among these 10 genes, four heat shock proteins (HSP; *HSPH1*, *HSPA4L*, *DNAJA4*, and *HSP90B1*) were identified as part of the network, interacting not only with each other but also with four other genes. Two other genes showed an interaction: C20orf96 and TNNT3, however, but they did not interact with the HSPs.

Considering the ten genes included in the network and based on the outcomes of the DAVID bioinformatic resource (see “Methods”), two terms were overrepresented within the set of genes: protein processing in endoplasmic reticulum (P = 0.003, Benjamini adjusted P = 0.013) and cytosol (P = 0.006, Benjamini adjusted P = 0.12). Looking at individual annotation terms provided by DAVID, the four HSPs had terms such as protein folding and refolding, protein processing in endoplasmic reticulum, endoplasmic reticulum chaperone complex, among others (Table 1). Additionally, and considering that the gene C20orf96 does not have a functionality yet discovered, another bioinformatic resource was used to look for other potential interactions between this DEG and other proteins. The STRING database (Search Tool for the Retrieval of Interacting Genes/Proteins) was used to search for other proteins, using “Gallus gallus” as the organism. This tool revealed only one link between C20orf96 and another protein, Aprataxin (APTX; considering that this link was not learnt using BN, the link is not part of Fig. 1).

**Table 1:** Functional Annotation Table provided by the Database for Annotation, Visualization, and Integrated Discovery (DAVID) corresponding to the Heat Shock Proteins interacting with other four genes.

**DnaJ (Hsp40) homolog, subfamily A, member 4 (***DNAJA4***)**	
GO TERMS	Response to heat, **protein refolding,** negative regulation of inclusion body assembly, cytosol, membrane, ATP binding
**Heat shock 105 kDa/110 kDa protein 1 (*HSPH1*)**	
KEGG PATHWAY	**Protein processing in endoplasmic reticulum**
**Heat shock 70 kDa protein 4-like (*HSPA4L*)**	
GO TERMS	Cytosol, ATP binding
KEGG PATHWAY	**Protein processing in endoplasmic reticulum**
**Heat shock protein 90 kDa beta (Grp94), member 1 (*HSP90B1*)**	
GO TERMS	response to hypoxia, **protein folding**, intracellular sequestering of iron ion, **response to stress**, ER-associated ubiquitin-dependent protein catabolic process, retrograde protein transport, ER to cytosol, actin rod assembly, negative regulation of apoptotic process, regulation of phosphoprotein phosphatase activity, cellular response to ATP, nucleus, **endoplasmic reticulum**, endoplasmic reticulum lumen, endoplasmic reticulum membrane, cytosol, plasma membrane, focal adhesion, midbody, extracellular matrix, **endoplasmic reticulum chaperone complex**, perinuclear region of cytoplasm, extracellular exosome
KEGG PATHWAY	**Protein processing in endoplasmic reticulum**

In bold are highlighted particularly relevant terms to the stress condition.

## Discussion

The complexity of biological systems generates a wealth of data that can be analysed in many ways; here we explored gene expression studies in chickens, focusing on the stress response. In the current study, we took a series of steps to identify an initial stress signal by using bioinformatic analysis, followed by the augmentation of the data with other datasets sharing the same animal model, the same tissue, and the same high-throughput technology. Thereafter, the datasets were discretized into discrete state variables; and finally, the structures of the BNs were learnt, revealing interactions among genes related to stress.

Initially, bioinformatic analyses allowed us to identify the stress signal, represented by the 25 genes showing differential expression patterns between the control and the heat stress conditions. We selected this dataset as the starting point for our study in order to minimise the complexity of the initial assumptions in our explanation of the data, and to parallel potential situations of researchers with their own pilot data. To gain greater statistical power when exploring interactions amongst these genes, we augmented the dataset with other experiments.

Augmenting the dataset also allowed us the possibility of exploring the stress phenomenon from a broader perspective, complementing previous studies, instead of independently focusing on them. Additionally, this enabled us to explore how these genes interact, not limited by experimental design: the initial stress experiments identified the genes of interest, the additional datasets provided further data on how these interact within the organism. After learning the structure of the BNs, 10 genes displayed informative and functional interactions. In this sense, taking a further step into a genetic system can shine further light onto the hidden patterns behind it. Using bioinformatic tools plus the power of BNs can identify informative genetic features, avoiding the extensive lists of genes related to numerous aspects of the physiology of the organism, such as immunity, cell signalling, neurogenesis, hormones, among many others. Different studies using only bioinformatic tools identified over a thousand genetic features between two conditions, such as domestic vs ancestral chickens, an immune challenge, or even stress, and the underlying biological mechanisms were varied in nature, pointing towards receptors (such as GABA), channels, steroidogenesis, the immune system (involving biological processes, cellular components, and molecular functionality), pathways such as signal transduction, enzymatic processes, and transcription activities^8,9,12,13^.

Among the 10 DEGs that displayed functional and informative relationships, four of them were HSP, and they displayed interactions not only between themselves, but also with four other genes. These interactions between the HSP and *BAG3*, *RNPC3*, *CRELD2*, and *XPO1*, might be closely related to the biological functionality of these proteins. HSPs are involved in stress tolerance and resistance, playing an important role in protecting the structure of other proteins such as enzymes or receptors, maintaining their correct structure, and consequently, their functionality^14,15^. During a stress event, especially under the influence of high environmental temperatures, the gene expression levels of these proteins are increased in several tissues such as brain, liver, lungs, heart, and breast^14,15^. It is then plausible to think that the unravelled interactions discovered by BNs are in agreement with previous studies, with the main difference being that, in our study, the relationships were identified from an initial stress signal, and then augmented. The other two genes, *C20orf96* and *TNNT3* (troponin T3, fast skeletal type), displayed an interaction between one another, but did not interact with the HSPs. *C20orf96* is an open reading frame conserved in human, mouse, chicken, and other animal models, but its functionality as a gene is yet unknown. As an additional pieces of evidence, the STRING database revealed a protein–protein interaction with Aprataxin (APTX)^16^. According to STRING, this APTX protein plays a role in repairing single-strand and double-strand DNA break as well as base excision, sometimes induced by reactive oxygen species. On the other hand, *TNNT3* is a member of the tropomyosin family, and it has been reported to play an important role in regulating the growth of dendritic cells in the nervous system of *Drosophila*, in close association with another gene, “*flamingo*”^17^. Based on the exploratory nature of learning the structure of BNs from the data, it is important to highlight the value of this identified relationship between *C20orf96* and *TNNT3*, as it represents a subject for the development of further studies aimed at studying the genetic implications of the functional interactions with *TNNT3*, *APTX* and *flamingo*.

The DAVID bioinformatic tool was used with the aim of gathering different pieces of information about the 10 DEGs, such as KEGG pathways, Gene Ontology (GO) terms, and overrepresented terms. Two terms were overrepresented within the set of genes: protein processing in endoplasmic reticulum (P = 0.003, Benjamini adjusted P = 0.013) and cytosol (P = 0.006, Benjamini adjusted P = 0.12). Even though HSPs can be found outside the cell, potentially as stress signals, their biological functions are mostly developed inside the cell^14,15^. The interactions discovered by the implementations of a BN approach between HSPs and the other four genes can be closely associated with the role of these proteins protecting the structures, and therefore, maintaining the correct functioning of other cytosolic proteins^14,15^. Considering the individual annotation terms, the HSPs had terms such as protein folding and refolding, protein processing in endoplasmic reticulum, endoplasmic reticulum chaperone complex, among others. Taking into consideration the biological functions of HSPs, the unravelled relationships and interactions between these four genes and the HSPs might be relevant to the physiology of birds, especially during stress conditions, when maintaining the optimum functioning of the cell machinery would be crucial to deal with the stressor^14,15^. Although the overrepresented terms might be slightly biased due to the relatively small set of DEGs, the information gathered is biologically coherent^18^. Additionally, nine out of ten DEGs had a proper annotation, and four out of those nine were HSP. It is, in a sense, expected that they share common terms, such as cytosol and protein processing in the endoplasmic reticulum, bearing in mind the previously discussed functionalities of HSPs. Considering that working with high-throughput technologies has a similar approach to knowledge discovery and big data, there should always be a balance between the bioinformatic aspects of the approach while considering the biology behind the results^18,19^.

Considering that the data came from experiments measuring gene expression in the brain, the functionality and activity of these HSPs can be understood as brain specific. However, as mentioned before, previous studies suggest that HSPs are expressed in different tissues in response to stress. Guo et al. reported that the exposure to stress (addition of CORT to the diet) differentially affected the gene expression of HSP such as HSPA2, HSPA8, HSP90AA1, and HSPH1 (the latter one also discovered in our study)^20^. Xie et al. also found differences in the expression patterns of two of the most common HSPs, HSP70 and HSP90, in the muscle, heart, and liver when laying hens were exposed to acute and chronic heat stress^21^. Although HSPs are expressed during a heat stress event, other stressors can also affect the expression of these proteins. Najafi, Zulkifli, and Soleimani found that higher expression levels of HSP70 were increased in the brain of chickens exposed to a feed restriction regimen^22^. Najafi et al. also evaluated the effects of stocking density on the same HSP70, and higher values were found in broilers bred under high stocking densities^23^. Al-Aqil et al. also found higher levels of the same HSP after 3 h of exposing chicks to transportation^24^. Our stress signal was initially found between control and heat stressed chickens, while the data augmentation involved other databases, some of them evaluating other types of stressors. The presence of four HSPs in the BN might indicate that, although the databases did not share the same stressor, HSP interactions within multiple types of stress were still strong and robust.

This study highlights the importance of interdisciplinary approaches to complex biological problems. Initially, bioinformatics was used to identify a set of genes with differential expression patterns, followed by the application of BNs as a machine learning tool to unravel the relationships between a small set of genes, and finally, the implementation of publicly available online resources for integrated discovery to understand the biological meaning of the unravelled interactions. It is important to emphasize the power of BNs in discovering and unravelling the relationships among a given set of genes from the data themselves^10^. The initial step of learning the structure of the network involved a set of 25 genes, but only 10 of them were part of the overall structure of the network. Interestingly, even though the remaining 15 DEGs showed differential expression patterns driven by the stress condition, they did not show interactions between them. The exploratory nature of BN allowed us the possibility of identifying the interaction between two of the DEGs, one of which does not have a functionality yet discovered. The DEG interacting with *C20orf96* codes for a protein belonging to the tropomyosin family^17^, and this information extracted from the BN itself can be used to design future studies in order to evaluate both the functionality and the potential interaction. This fact also highlights that an exploratory approach with BNs can be implemented in knowledge discovery.

Learning the structure of a BN is not an easy road, as it involves multiple steps to be taken and decisions to be made. Even though the initial dataset used for identifying genes associated with stress had enough individuals to perform bioinformatic analysis with the aim of identifying genes with differential expression patterns driven by the exposure to the stress condition, it represented a small number for learning BNs. However, in our study, we overcame this challenge by augmenting the initial dataset with four other studies: the topmost highly significant DEGs were tracked to other datasets, despite the complexity of their experimental design, with the aim of increasing the number of observations. Choosing the best approach to collecting, re-using, and combining multiple datasets varied in nature was the first challenge we faced. As with the following step, the discretization of the data, there are multiple pathways to choose from. Two previous studies in genetics and epigenetics combined two databases into one larger database, but the main difference is that they shared a common stressor, either heat stress or social isolation^25,26^. The structure of the BNs included the stress condition as a node, and some genetic/epigenetic variables were directly interrelated to stress^25,26^. However, in our case, due to the complexity of the experimental designs, it was not possible to include stress as another node. The initial stress signal was identified in a simple but effective experimental design, evaluating the effects of stress in a set of chickens under either control or stress conditions. Thereafter, we searched for other databases sharing the same animal model, same tissue, and same high-throughput technology, as a way to increase the robustness of the BN searches as well as the statistical/computational power. And to deal with the challenge of considering different experimental designs and the potential noise introduced by each particular experimental design, each dataset was discretized into three-state variables before building the final dataset^27^. Previous studies have discussed the advantages and disadvantages of discretising a database, but there must be a balance between gaining statistical power, dealing with multiple experimental designs, and the loss of information.

To conclude, a multidisciplinary approach was implemented to reduce the initial number of genes obtained from high-throughput technologies to a small number of DEGs, followed by the discovery of relationships and interactions between the DEGs. The approach involved the combination of bioinformatics, BNs, and two bioinformatic resources: DAVID and STRING, as resources for biological knowledge discovery. The overall results showed that four HSPs have interactions not only between themselves but also with four other genes, potentially highlighting their biological functions to protect the structure and to maintain the correct functioning of proteins within the cell. Considering the exploratory nature of our study, future research can be oriented to determine the discovered protein–protein interactions*,* evaluating the differences between chickens raised under control conditions and chickens raised under stress conditions.

## Methods

**Figure 2:**
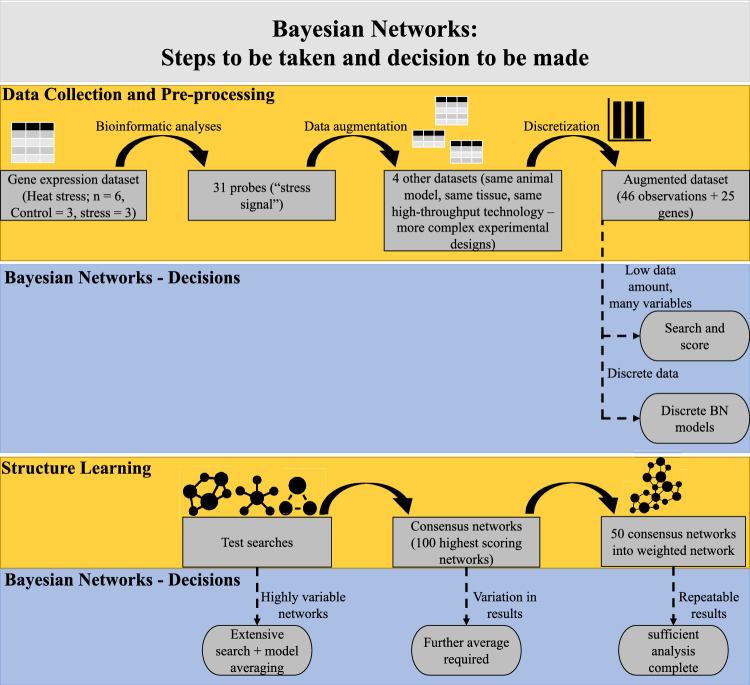
Bayesian networks: Steps to be taken and decisions to be made. The starting point was to identify a set of differentially expressed genes (DEGs) between chickens raised under control (n = 3, temperature 28 ± 1 °C) and raised under heat stress conditions (n = 3, temperature 40 ± 1 °C, for 3 h). These 31 probes were considered as our initial stress signal, using an adjusted P-value equal to or lower than 0.02. Considering that the number of observations was borderline sufficient to learn useful structure of BNs, we searched this stress signal represented by these 31 probes in other four databases, sharing the same animal model (the chicken), the same tissue (the brain), and the same high-throughput technology (microarray). The average expression value was taken for those probes coding for the same gene, giving a total of 25 DEGs. Before learning the structure of the network, the datasets were individually discretized into three-state variables using a quantile discretization method, and then combined into a larger dataset consisting of 46 observations and 25 DEGs. The software Banjo was used to learn the structure of the BNs, visiting a total of 250 million networks, scoring them with the BDe score, using a Simulated Annealing algorithm. The consensus BN was built with the top 100 scoring networks. After running the algorithm several times, the searches were slightly unstable, showing small differences in the set of arcs. Therefore, 50 consensus BNs were overlapped into a weighted network, and a final resulting network generated by selecting those arcs present in at least 50% of the searches (25 networks out of 50). The process was repeated four times, to make sure of the reproducibility of our results.

Figure 2 shows the steps taken and the decisions made to collect and augment the data, learn the structure of the networks, and combine multiple searches into one weighted network, following the patterns of our previously published roadmap for such analyses^25^.

### Dataset

Initially, differentially expressed genes were identified in a publicly available gene expression dataset, consisting of data from six samples, three of them coming from chickens reared under control conditions, while the other three were exposed to heat stress (GEO accession number GSE23592). According to the experimental design, chickens were assigned either to a control treatment (temperature 28 ± 1 °C) or to a heat stress treatment (temperature 40 ± 1 °C, for 3 h), and gene expression was measured by microarray technology on brain samples. To select this dataset as the starting point of our study, one of the premises of machine learning and statistical modelling was slightly modified to suit our initial goal: the first option should always be the simplest model. In our study, we translated this premise into the experimental design, choosing the study with a simple yet effective experimental design. The R package “*affy*”^28^ was implemented to pre-process the files, to normalise, and to correct the background noise. The R package “*limma*”^29^ was implemented to extract the expression values, as well as to fit a linear model to identify and then select probes corresponding to a set of differentially expressed genes (DEG). A total of 1397 probes were initially identified as differentially expressed by applying the function “*topTable*”, considering the adjusted P-value (false discovery rate) provided by the same function to be less than or equal to 0.05. However, considering that the number of DEGs was relatively high to implement BNs, after examination of the P-value distribution (see Supplementary Fig. S1 online), an adjusted P-value less than or equal to 0.02 was selected as a threshold to select the topmost highly significant DEGs. This cut-off constrained the number of significant probes to 31 for further analysis. The initial number of individuals was enough to identify DEGs, but it was not enough to implement our BN algorithms^30^, therefore, these identified DEGs were traced to another four datasets, sharing three characteristics: the same animal model (chickens), the same tissue (brain—thalamus/hypothalamus), and the same high-throughput technology (microarrays) (ArrayExpress accession numbers: E-MTAB-924, E-MTAB-3319, E-MTAB-644, and E-MTAB-645). Due to the complexity of the experimental designs of these four databases, it was not possible to consider the stress condition as a variable. Although these other four studies evaluated stress in a way, such as social isolation, physical restraint, or high/low fear response to humans, they also considered other factors, such as sex, age, or breeds (red junglefowl/white leghorns). The expression values for each gene were extracted after normalizing and correcting the background noise, using the R package “*affy*”. Thereafter, the 31 probes were annotated, and probes coding for the same gene (12 probes coded for 6 different genes, 6 genes × 2 probes = 12) were averaged into one single value, representing the mean between the two expression values for each pair of observations. Before merging the five datasets into one larger dataset, and considering that each dataset evaluated different experimental designs, each individual dataset was discretized into three categories, using a quantile discretization method, to remove potential noise^27^. The R package “*arules*” was used to achieve this aim, with the "*discretizeDF*” function, the “frequency” method, and three breaks (categories)^31^. The three categories were low, medium, or high, and each one of them had the same number of observations per category (when possible). As a result, the initial stress signal was augmented by four other studies, and a final dataset consisting of 25 genes and 46 individuals was used for further analysis.

### Bayesian networks

BNs were learnt in Banjo, available for free for academic purposes from http://www.cs.duke.edu/~amink/software/banjo/. The algorithm implemented in this study was Simulated Annealing, exploring the search space from an empty graph, and scoring each network with the BDe score. Banjo allows the possibility to adjust the search parameters; in this particular study, the search space was explored with a total of 250 million networks with local random moves as the proposer. BN algorithms search the space based on a given set of variables, adding, removing, or reversing arcs. After each one of these changes, the new network is scored, and its score is compared to the score corresponding to the previous network. In Simulated Annealing, if the score of the new network is higher than the previous network, the latter is discarded; if the score is lower, the probability of accepting the new network is based on a temperature parameter which slowly reduces this probability as the search proceeds. The process is iterated, with the slowly reducing temperature parameter, until only networks with higher scores are accepted; at this point the search continues until no further improvements in the score metric can be made^10,32^.

Considering that the process is based on heuristic random searches, different searches had slight differences in the final set of arcs. Banjo can also build a consensus network, providing only arcs that have a probability of being present of at least 50%, based on a selection of top-scoring networks. To address variation in arcs, a consensus network was built in Banjo combining the top 100 high-scoring networks. However, there was still variation even in these consensus networks. Therefore, weighted networks were built by combining the results of 50 consensus networks into a matrix of presence/absence: for each individual network, if an edge was present a value of one was assigned, while if the edge was absent, a value of zero was assigned. The values for each edge were summed across the 50 networks to generate a weighted network representing the number of consensus networks containing that edge; the resulting edge weights ranged from 1 to 50. Those arcs present in at least 50% of the consensus networks (in 25 or more networks) were used to construct a final network showing arcs with the most support. To evaluate the consistency of this approach, it was repeated four times, and there were no differences in the structure of the resulting final networks (each final network consisted of the same set of arcs).

### Biology behind the results

To provide further insights into the biological meaning, the database for annotation, visualization, and integrated discovery (DAVID) was explored^33^. DAVID is a bioinformatic resource, publicly available (https://david.ncifcrf.gov), that combines different sources of information, such as KEGG pathways^34^, protein–protein interactions, bio-pathways, GO terms, homology, among many others, with the aim of closing the gap between a list of statistically significant genes and their functionality as well as their biological meaning^33^. The genes that were part of the weighted network were used to find annotation terms significantly overrepresented among the list of genes as a whole as well as the individual annotation terms for each of the genes. These pieces of information are found in the Functional Annotation Chart and the Functional Annotation Table, respectively^33^. The list of genes was included in the “Upload” tab with the Affymetrix microarray option, and Chicken Array—Gallus gallus were used as “Background”. In addition to DAVID, the STRING database (Search Tool for the Retrieval of Interactive Genes) was also used to further understand the biology behind one of the DEGs (C20orf96) that did not have a functionality yet discovered. This database is available for free (https://string-db.org), and it provides known and predicted protein–protein interactions, allowing the possibility to find potential genes in close association with our gene of interest^16^. This gene, C20orf96, was used as the protein name, and *“Gallus gallus domesticus”* was selected as the organism.

### Supplementary Information

Supplementary Figure S1.

Supplementary Legends.

Supplementary Table S1.

## Data Availability

The datasets analysed during the current study are available in the GEO repository, accession number GSE23592, and the Array Express repository, accession numbers E-MTAB-924, E-MTAB-3319, E-MTAB-644, and E-MTAB-645.

## References

[1] De Kloet ER (2003). Hormones, brain and stress. Endocr. Regul..

[2] McEwen BS (1997). The role of adrenocorticoids as modulators of immune function in health and disease: Neural, endocrine and immune interactions. Brain Res. Rev..

[3] Siegel HS (1971). Adrenals, stress and the environment. Worlds Poult. Sci. J..

[4] Kumar M, Ratwan P, Dahiya SP, Nehra AK (2021). Climate change and heat stress: Impact on production, reproduction and growth performance of poultry and its mitigation using genetic strategies. J. Therm. Biol..

[5] Cantet JM, Yu Z, Ríus AG (2021). Heat stress-mediated activation of immune–inflammatory pathways. Antibiotics.

[6] Elfwing M (2015). Early stress causes sex-specific, life-long changes in behaviour, levels of gonadal hormones, and gene expression in chickens. PLoS One.

[7] Bélteky J, Agnvall B, Johnsson M, Wright D, Jensen P (2016). Domestication and tameness: Brain gene expression in red junglefowl selected for less fear of humans suggests effects on reproduction and immunology. R. Soc. Open Sci..

[8] Fallahsharoudi A (2015). Domestication effects on stress induced steroid secretion and adrenal gene expression in chickens. Sci. Rep..

[9] Pértille F (2020). Putative epigenetic biomarkers of stress in red blood cells of chickens reared across different biomes. Front. Genet..

[10] Heckerman D, Geiger D, Chickering DM (1995). Learning Bayesian networks: The combination of knowledge and statistical data. Mach. Learn..

[11] Nagarajan R, Scutari M, Lèbre S (2013). Bayesian Networks in R: With Applications in Systems Biology.

[12] Kuchipudi SV (2014). Highly pathogenic avian influenza virus infection in chickens but not ducks is associated with elevated host immune and pro-inflammatory responses. Vet. Res..

[13] Pértille F (2017). DNA methylation profiles in red blood cells of adult hens correlate with their rearing conditions. J. Exp. Biol..

[14] Goel A, Ncho CM, Choi YH (2021). Regulation of gene expression in chickens by heat stress. J. Anim. Sci. Biotechnol..

[15] Perini F (2021). Emerging genetic tools to investigate molecular pathways related to heat stress in chickens: A review. Animals.

[16] Szklarczyk D (2021). The STRING database in 2021: Customizable protein-protein networks, and functional characterization of user-uploaded gene/measurement sets. Nucleic Acids Res..

[17] Li W, Gao FB (2003). Actin filament-stabilizing protein tropomyosin regulates the size of dendritic fields. J. Neurosci..

[18] Wijesooriya K, Jadaan SA, Perera KL, Kaur T, Ziemann M (2022). Urgent need for consistent standards in functional enrichment analysis. PLoS Comput. Biol..

[19] Greene CS, Tan J, Ung M, Moore JH, Cheng C (2014). Big data bioinformatics. J. Cell. Physiol..

[20] Guo Y (2020). Identification of genes related to effects of stress on immune function in the spleen in a chicken stress model using transcriptome analysis. Mol. Immunol..

[21] Xie J (2014). Differential expression of heat shock transcription factors and heat shock proteins after acute and chronic heat stress in laying chickens (Gallus gallus). PLoS One.

[22] Najafi P, Zulkifli I, Soleimani AF (2018). Inhibition of corticosterone synthesis and its effect on acute phase proteins, heat shock protein 70, and interleukin-6 in broiler chickens subjected to feed restriction. Poult. Sci..

[23] Najafi P (2015). Environmental temperature and stocking density effects on acute phase proteins, heat shock protein 70, circulating corticosterone and performance in broiler chickens. Int. J. Biometeorol..

[24] Al-Aqil A (2013). Changes in heat shock protein 70, blood parameters, and fear-related behavior in broiler chickens as affected by pleasant and unpleasant human contact. Poult. Sci..

[25] Videla Rodriguez EA (2022). Practical application of a Bayesian network approach to poultry epigenetics and stress. BMC Bioinf..

[26] Videla Rodriguez EA, Mitchell JBO, Smith VA (2022). A Bayesian network structure learning approach to identify genes associated with stress in spleens of chickens. Sci. Rep..

[27] Balov N (2013). A categorical network approach for discovering differentially expressed regulations in cancer. BMC Med. Genom..

[28] Gautier L, Cope L, Bolstad BM, Irizarry RA (2004). Affy—Analysis of Affymetrix GeneChip data at the probe level. Bioinformatics.

[29] Ritchie ME (2015). Limma powers differential expression analyses for RNA-sequencing and microarray studies. Nucleic Acids Res..

[30] Yu J (2005). Developing Bayesian Network Inference Algorithm to Predict Casual Functional Pathways in Biological Systems.

[31] Hahsler M, Grün B, Hornik K (2005). Arules—A computational environment for mining association rules and frequent item sets. J. Stat. Softw..

[32] Liu J, Bo S (2011). Naive Bayesian classifier based on genetic simulated annealing algorithm. Procedia Eng..

[33] Huang DW, Sherman BT, Lempicki RA (2009). Systematic and integrative analysis of large gene lists using DAVID bioinformatics resources. Nat. Protoc..

[34] Kanehisa M, Goto S (2000). KEGG: Kyoto encyclopedia of genes and genomes. Nucleic Acids Res..

