# Supplementary material for: Robust identification of interactions between heat-stress responsive genes in the chicken brain using Bayesian networks and augmented expression data
Source: Sci Rep. 2024 Apr 19;14:9019. doi: 10.1038/s41598-024-58679-3 (PMC11031576; doi:10.1038/s41598-024-58679-3)
Supplement: Supplementary file 1 — Supplementary Figure S1. [file 41598_2024_58679_MOESM1_ESM.pdf]

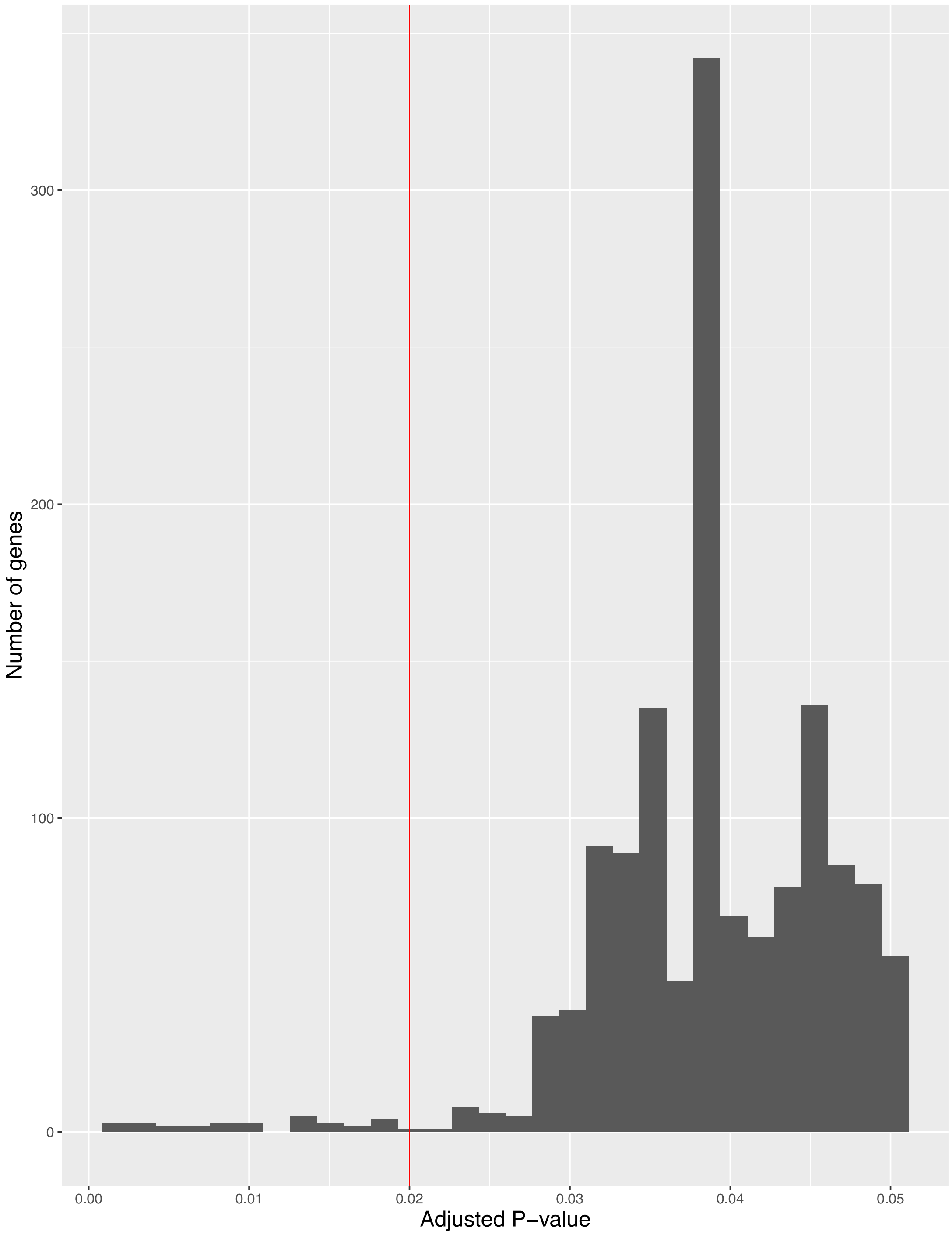

Supplementary Figure S1. **Adjusted p-value distribution of differentially expressed probes.** Bar plot corresponding to the adjusted P-values of highly significant probes identified by bioinformatic tools. The x-axis represents the adjusted P-values, the y-axis represents the number of observations. The red solid line represents the threshold used to select the 31 probes coding for the 25 differentially expressed genes, initially identified as the stress signal. An adjusted p-value of 0.02 (red line) was selected as capturing a reasonable tail of the p-value distribution. The threshold used retrieved a manageable number of probes to further explore the complex biological system with Bayesian networks.
