# Supplementary material for: Robust identification of interactions between heat-stress responsive genes in the chicken brain using Bayesian networks and augmented expression data
Source: Sci Rep. 2024 Apr 19;14:9019. doi: 10.1038/s41598-024-58679-3 (PMC11031576; doi:10.1038/s41598-024-58679-3)
Supplement: Supplementary file 2 — Supplementary Legends. [file 41598_2024_58679_MOESM2_ESM.docx]

**Supplementary files**

Supplementary Table S1

Excel file .csv

Differentially expressed genes

List of those 25 genes identified as differentially expressed from the publicly available dataset on heat stress in chickens.

Supplementary Figure S1

Portable document format .pdf

Adjusted p-value distribution

An adjusted p-value of 0.02 (red line) was selected as capturing a reasonable tail of the p-value distribution.
